# Correlation of clinical, cytological and histological findings in oral squamous cell carcinomas

**DOI:** 10.3892/ol.2014.2212

**Published:** 2014-06-03

**Authors:** MICHELE CARDOSO SOUSA, MONICA GHISLAINE OLIVEIRA ALVES, LUCIANO ALBINO SOUZA, ADRIANA AIGOTTI HABERBECK BRANDÃO, JANETE DIAS ALMEIDA, LUIZ ANTONIO GUIMARÃES CABRAL

**Affiliations:** Department of Biosciences and Oral Diagnosis, Institute of Science and Technology, UNESP, Univ. Estadual Paulista, São José dos Campos, São Paulo 12245-000, Brazil

**Keywords:** pathology, squamous cell carcinoma, oral cancer, cytology

## Abstract

The present study aimed to investigate the efficiency of exfoliative cytology by correlating the clinical lesions of oral squamous cell carcinoma (OSCC) with exfoliative cytology and histopathological findings. Cases of OSCC diagnosed between 1984 and 2010 were analyzed. The inclusion criteria for the present study were the availability of detailed clinical findings and a diagnosis of the disease through exfoliative cytology and histopathology. The cases were assessed and assigned scores, which were then submitted to modal expression analysis, which considers the higher frequency scores, thus relating the variables. The cytological findings demonstrated that the majority of the cases had malignant potential. Exfoliative cytology should be used as a supplementary tool for the diagnosis of OSCC, as it enables the early detection of these lesions. However, cytology should not be used as a substitute for histopathological examination.

## Introduction

Oral squamous cell carcinoma (OSCC) is the most common type of cancer of the oral cavity worldwide (1–3). In Brazil, the annual number of cancer-associated mortalities is 6,214, with ~14,120 news cases reported in 2010. OSCC is the sixth most common type of cancer and the most frequent type of head and neck cancer (4). The disease primarily affects males between 40 and 65 years old (2). Smoking is the most significant etiological factor for the development of OSCC, and the risk of OSCC increases markedly if smoking is combined with alcohol consumption (5–7). The lateral border of the tongue is the most affected oral site (2,8). In patients with OSCC, survival is directly associated with early diagnosis (6,9), particularly in those with a potentially malignant disorder, including leukoplakia and erythroplakia, which may precede the development of OSCC or be present in association with OSCC (10).

In the early stages, OSCC frequently clinically manifests as inoffensive with asymptomatic lesions. As a consequence, patients are likely to postpone medical care, thus delaying the diagnosis and adequate treatment, resulting in a poorer prognosis (11,12). By contrast, the difficulty in establishing an accurate diagnosis is lower in cases in which the symptoms are more pronounced (3). Anatomopathological examination of lesion biopsies is the most important method for diagnosing OSCC (3,13,14). However, obtaining a sample through biopsy is invasive and technically difficult (15). Exfoliative cytology has been shown to be an efficient diagnostic method, particularly in the more advanced stages of the disease (13), and cytological analysis is beneficial for assessing cellular alterations in epithelial tissues exhibiting a normal appearance (16).

Raab and Grzybicki (17) proposed that correlation analyses are highly valuable in the fields of cytopathology and surgical pathology, as correlation analysis generates much data that may be used to improve diagnostic testing and screening processes. However, it is necessary to develop standardized methods for correlation analyses and to use correlation data to redesign testing and screening processes to enhance the quality of such processes, as well as patient safety (17).

The present study aimed to correlate the clinical lesions of OSCC with exfoliative cytology and histopathological findings in order to assess the efficiency of exfoliative cytology.

## Materials and methods

### Patients

The present study was approved by the Ethics Committee of Institute of Science and Technology, UNESP, Univ. Estadual Paulista (São José dos Campos, Brazil; protocol no. 044/2009-PHCEP) and patients provided written informed consent. Cases of OSCC that were diagnosed at the Institute of Science and Technology, UNESP, Univ. Estadual Paulista between 1984 and 2010 were analyzed. The clinical, histological and cytological records of patients observed at the Stomatology outpatient clinic were reviewed for data collection.

Patients were included in the present study based on the following inclusion criteria: (i) The availability of detailed clinical findings; and (ii) the diagnosis of OSCC using exfoliative cytology and histopathology. Scores were attributed to the morphological features of each case. The clinical findings are presented based on the degree of aggressiveness of the OSCC (Table I).

## Exfoliative cytology

Patients underwent exfoliative cytology of the lesion using a cytobrush (Vagispec, Jaraguá do Sul, SC, Brazil), and all samples were stained using the Papanicolaou method. The results were classified with using the following criteria proposed by Papanicolaou and Trout (18) (Table II): Class I, normal; class II, inflammatory changes; class III, atypical cells, suspect smear; class IV, non-conclusive of malignancy; and class V, malignant.

### Histological staining

The histological sections obtained from the patients with OSCC were stained using hematoxylin and eosin, and were analyzed using light microscopy by an examiner. Slides were scored between 1 and 4 according to the system proposed by Anneroth *et al* (19) (Table III).

For better correlation, the results were submitted to modal expression using the scores, considering the higher frequency scores related to each other.

## Results and Discussion

In total, 53 of the 316 OSCC cases met the inclusion criteria; 41 were male and 12 were female, with an age range between 28 and 88 years. Table IV shows the correlation between the clinical, cytological and histological findings. Among the 53 cases analyzed, 28.3% were classified as Papanicolaou class III, 3.77% as class IV and 58.71% as class V. Thus, 86.79% of the cases were found to have malignant potential.

Previous studies that have compared the efficacy of exfoliative cytology and histopathology for the diagnosis of OSCC have shown that exfoliative cytology is an effective method (1,13,20). In the present study, only 53 cases of OSCC met the inclusion criteria. The majority of these cases were from the Stomatology Outpatient Clinic of the Institute of Science and Technology, indicating that exfoliative cytology may be primarily used at universities. Although this technique is practical, inexpensive, simple and non-invasive (3,13,21), dentists do not use this method routinely for the early detection of OSCC (14,22).

The results of the present study showed no direct correlation between the clinical, cytological and histological scores. This finding indicates that it is not possible to predict the behavior of OSCC based solely on the observation of clinical features. In accordance with this, in the present study, a histological score of 2 or 3 was attributed to three cases of erythroleukoplakia, demonstrating that the mild clinical findings did not correspond with the aggressive histological characteristics. In addition, a histological score of 1 was attributed to one case presenting a nodule that was clinically scored as 2.

In the present study, three cases showing clinical features of a shallow ulcer <2 cm, exulceration >2.5 cm and a destructive and infiltrative ulcer were classified as Papanicolaou class I or II using cytological analysis. These findings, which demonstrate the lack of efficiency of exfoliative cytology, may be explained by non-representative sampling and/or individual subjectivity (17,23), since this is a retrospective study in which the cytological tests were not performed by the same examiner.

Exfoliative cytology involves the analysis of superficial epithelial cells that are obtained through scraping (24,25) the lesion using a sterile cytobrush (22). Thus, collecting deeper cells from plaques or nodules is difficult, which compromises the accuracy of the technique (1,15). This limitation was observed in the present study, in which three cases presenting with nodules and one case presenting with an erythroleukoplakia plaque were diagnosed as cytological score 1. In the present study, analysis of the cytological findings suggested that the majority of the cases had malignant potential, indicating that exfoliative cytology may be beneficial as a supplementary tool for diagnosing OSCC.

Oral cytology is useful for monitoring patients undergoing treatment in order to guide the selection of sites for incisional biopsies (26,27) and to analyze lesions with malignant potential with high sensitivity and specificity, leading to an early diagnosis (28). In addition, oral cytology has been used to identify changes prior to their clinical visibility (27). The advantages of exfoliative cytology make it a particularly useful diagnostic method for obtaining early test results. Furthermore, cases with more symptoms that are associated with more advanced stages of the disease are easily diagnosed due to the obvious clinical features (29).

At present, dentists observe the development of non-specific ulcers for 14 days after the first visit, and then establish an objective diagnosis (30). Thus, exfoliative cytology may obtain a more rapid diagnosis (5,19,31).

Although exfoliative cytology should not be used as a substitute for histopathological examination, the present study has demonstrated the efficiency of exfoliative cytology for the diagnosis of OSCC and has shown that it may be beneficial as an additional tool to enable early referral of patients to a specialized service.

## Figures and Tables

**Table I:** Clinical findings according to the degree of the clinical aggressiveness of OSCC.

Erythroleukoplakia	Shallow ulcer or nodule ≤2.5 cm	Ulcerated or exulcerated lesion >2.5 cm	Destructive ulceroinfiltrative lesion
1	2	3	4

OSCC, oral squamous cell carcinoma.

**Table II:** Cytological findings classified according to the criteria proposed by Papanicolaou and Trout (18).

Class I and II	Class III	Class IV	Class V
1	2	3	4

Class I normal; class II, inflammatory changes; class III, atypical cells, suspect smear; class IV, non-conclusive of malignancy; and class V, malignant.

**Table III:** Histological findings according to the scoring system proposed by Anneroth *et al* (19).

	Score			
	
Morphological parameters	1	2	3	4
Degree of keratinization	High (>50% of cells)	Moderate (20–50% of cells)	Minimal (5–20% of cells)	Absent (0–5% of cells)
Nuclear polymorphism	Low (>70% of mature cells)	Moderate (50–75% of mature cells)	Abundant (25–50% of mature cells)	Maximal (0–25% of mature cells)
Number of mitoses	0–1	2–3	4–5	>5
Invasion pattern	Infiltrate with well-delimited borders pushing adjacent tissue	Cord-like infiltrate groups	Infiltrate of small groups (n>15) of cords	Infiltrate of small groups of cells (n>15), accompanied by cell dissociation
Stage of invasion (depth)	*In situ* carcinoma and/or potential invasion	Tissue invasion involving only the lamina propria	Invasion below the lamina propria and involving adjacent muscles, salivary glands and periosteum	Deep and extensive invasion replacing stroma and infiltrating mandibular bone
Inflammatory infiltrate	Abundant	Moderate	Mild	Absent

**Table IV:** Correlation between clinical, histological and cytological scores.

Cytological scores	Clinical score 1				Clinical score 2				Clinical score 3				Clinical score 4				Total
			
	H1	H2	H3	H4	H1	H2	H3	H4	H1	H2	H3	H4	H1	H2	H3	H4	
1	1	0	0	0	0	3	1	0	0	0	1	0	0	0	1	0	7
2	0	1	1	0	1	7	1	1	0	0	1	0	0	0	2	0	15
3	0	0	0	0	0	0	0	0	0	0	0	0	0	1	1	0	2
4	0	0	1	0	0	4	4	0	0	4	3	1	2	7	3	0	29
Total	4				22				10				17				53

H, histological.
